# Delta-Notch signalling in segmentation

**DOI:** 10.1016/j.asd.2016.11.007

**Published:** 2017-05

**Authors:** Bo-Kai Liao, Andrew C. Oates

**Affiliations:** a Francis Crick Institute, Mill Hill Laboratory, The Ridgeway, London NW7 1AA, UK; b Department of Cell and Developmental Biology, University College London, Gower Street, London WC1E 6BT, UK

**Keywords:** Somitogenesis, Clock and wavefront model, Doppler effect, Delta-Notch, Patterning, PSM, pre-somitic mesoderm, Fgf, fibroblast growth factor, Wnt, wingless int-1, DAPT, N-[N-(3,5-difluorophenacetyl-L-alanyl)]-S-phenylglycine t-butyl ester, Nrarp, Notch-regulated ankyrin repeat protein, 3D, three dimensional, bHLH, basic helix-loop-helix, DSL, Delta/Serrate/lag-2

## Abstract

Modular body organization is found widely across multicellular organisms, and some of them form repetitive modular structures via the process of segmentation. It's vastly interesting to understand how these regularly repeated structures are robustly generated from the underlying noise in biomolecular interactions. Recent studies from arthropods reveal similarities in segmentation mechanisms with vertebrates, and raise the possibility that the three phylogenetic clades, annelids, arthropods and chordates, might share homology in this process from a bilaterian ancestor. Here, we discuss vertebrate segmentation with particular emphasis on the role of the Notch intercellular signalling pathway. We introduce vertebrate segmentation and Notch signalling, pointing out historical milestones, then describe existing models for the Notch pathway in the synchronization of noisy neighbouring oscillators, and a new role in the modulation of gene expression wave patterns. We ask what functions Notch signalling may have in arthropod segmentation and explore the relationship between Notch-mediated lateral inhibition and synchronization. Finally, we propose open questions and technical challenges to guide future investigations into Notch signalling in segmentation.

## Highlights

• Vertebrate segmentation clocks are different to each other.
• Notch has a primary role in synchronization in vertebrates.
• Gene expression waves can influence the timing of segment formation.
• Synchronization and lateral inhibition circuits are closely related.
• Dynamic measurement techniques are needed to dissect clocks.

## 1 Introduction

Monitoring and predicting periodic events from the environment has evolutionary advantages. Therefore, cells and organisms have evolved various molecular machineries as biological clocks for determining time intervals. For example, circadian clocks contain entrainable oscillators with about 24-h rhythm for anticipating recurring daily activities. Even without modulatory signals from the environment, a circadian clock can still run by its endogenous machinery, but gradually loses synchrony with the external day–night rhythm (Golombek and Rosenstein, 2010).

Biological oscillators are not only used for coordinating with daily environmental factors, but have also been adopted to control periodicity of cellular or tissue events at higher frequency within organisms (Webb and Oates, 2016). Somitogenesis is a rhythmic process occurring in the presomitic mesoderm (PSM) in order to subdivide the undifferentiated tissue of the vertebrate embryo into the segments of the body axis, namely somites. The somites give rise to the segmented parts of the adult anatomy, namely the vertebrae, neural and hemal arches, ribs, and their associated muscles and overlying skin.

By studying avian embryos, Palmeirim et al. (1997) provided the first evidence that *c-hairy1*, an avian homologue of the *Drosophila hairy* gene, was expressed rhythmically in the PSM in a tissue-autonomous manner. Waves of rhythmic *c-hairy1* expression with 90-min period first appear in the posterior PSM, then travel anteriorly and finally arrest in the anterior PSM marking where each new somite boundary is defined (Palmeirim et al., 1997). Those findings provided the first evidence for the long-standing clock and wavefront hypothesis (Cooke and Zeeman, 1976) (see Section 2). The modern molecular version of this idea proposes that somitogenesis is driven by an oscillating multicellular genetic network termed the segmentation clock, and the term “cyclic gene” refers to those genes with expression resembling the *c-hairy1* wave patterns in PSM.

Several cyclic genes are found among the *hes*/*her* (*hairy* and *enhancer of split-related*) gene family in all species examined, suggesting that oscillation in this family is a conserved feature of the segmentation clock. In mouse, a single cyclic *hes*/*her* gene, *Hes7*, appears to play a central role in segmentation (Bessho et al., 2001a, Bessho et al., 2003). In zebrafish the roster includes *her1* (Holley et al., 2000), *her7* (Oates and Ho, 2002), proposed as components of the segmentation clock core pacemaking circuit in this species (Schröter et al., 2012). *her11* (Gajewski et al., 2006), *her12* and *her15* (Shankaran et al., 2007), *her2* and *her4* (Krol et al., 2011) have been shown to oscillate as well, but their role in the clock is not yet known. In contrast *hes6* was found not to be cyclically expressed in the zebrafish PSM based on mRNA spatiotemporal patterns (Kawamura et al., 2005, Schröter and Oates, 2010), yet the protein dimerises with other Her proteins and functions as a core component (Schröter et al., 2012, Trofka et al., 2012, Hanisch et al., 2013).

**Fig. 1:**
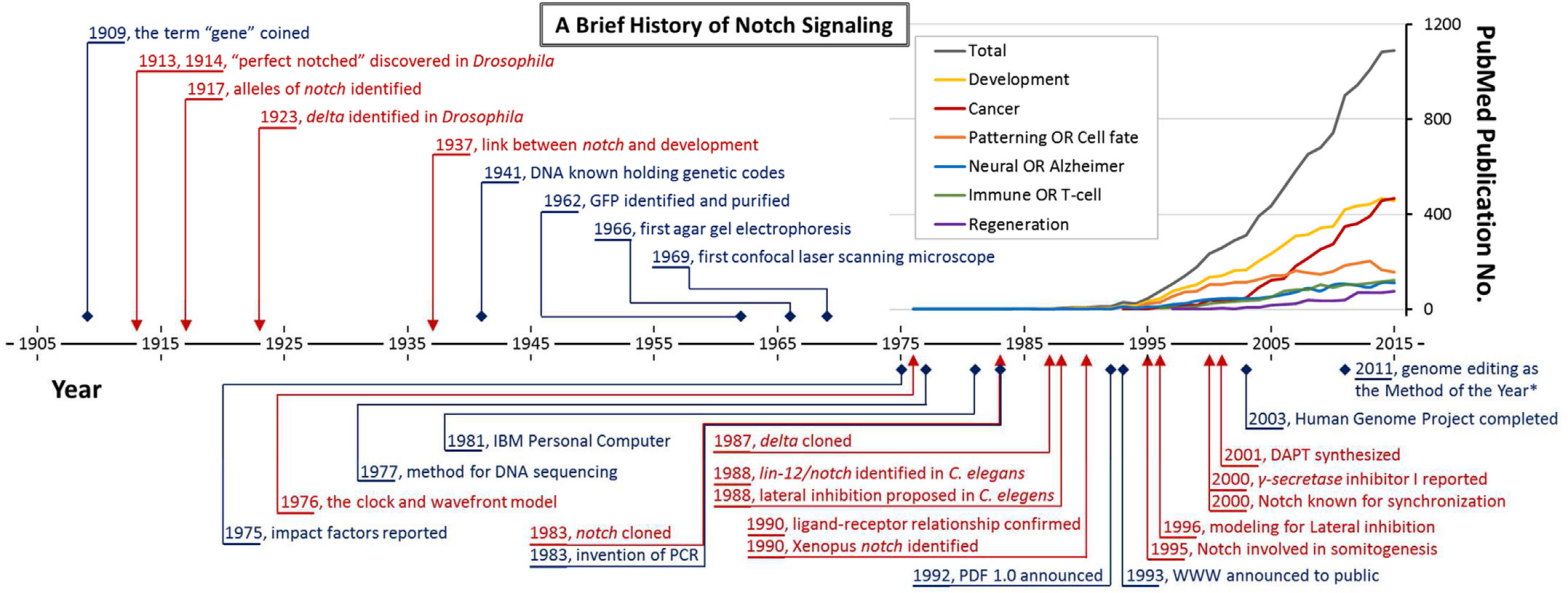
A brief history of Notch signalling. A simplified chronicle of selected important events about Notch signalling (red text) and the appearance of modern scientific tools (blue text) is illustrated in honour of the pioneers in the field. The inset at top-right corner is the publication numbers of Notch signalling from 1976 to 2015 in the PubMed database (http://www.ncbi.nlm.nih.gov/pubmed) searched with related keywords. The total (grey line) is searched by “notch signalling” OR “notch pathway” OR “delta notch”, and the sub-fields are AND search to the total with the keywords listed in the box. Two sub-fields have a different trend to the others; the cancer-related publications ascended faster than other others and the pattering/cell fate sub-field started to decline recently.

The second cyclic gene identified was *lunatic Fringe* (*lFng*) from chick and mouse (Evrard et al., 1998, Mcgrew et al., 1998). The *Fringe* family of genes encode glycosyltransferase enzymes that can modify sugar residues on Notch receptors, altering their binding preferences – this link between the segmentation clock and Delta-Notch signalling emerged due to previous discoveries from fruit flies (*Drosophila melanogaster*) showing that *Fringe* could alter Notch signalling (Fleming et al., 1997, Panin et al., 1997, Aulehla and Johnson, 1999, Dale et al., 2003). In contrast to mouse and chick, genes of the *Fringe* family do not oscillate in zebrafish (Prince et al., 2001), but other members of the Notch pathway do (see below). Notch has a relatively long scientific history in biology (Fig. 1) since it was first discovered more than a century ago (Morgan and Bridges, 1916, Morgan, 1917). The first publication of a *Notch* mutant was by Dexter (1914), who characterized the “perfect notched” phenotype on the wing edges of *Drosophila*. Nowadays, Notch signalling is one the most-studied signalling pathways, because it is versatile in biological function and also evolutionarily conserved in most laboratory model animals. The role of Notch signalling in segmentation is the main theme of this review.

The standard picture of Notch signalling involves ligands of the DSL (*Delta*/*Serrate*/*lag-2*) family on the surface of the signal-sending cell binding to Notch receptors on the receiving cell's surface. Notch receptors in the signal-receiving cell are processed in ER and Golgi to produce non-covalent heterodimers between the Notch Extracellular Domain (NECD) and the Transmembrane Domain-Notch Intracellular Domain (TM-NICD) by Furin based cleavage (S1 cleavage) before delivery to the plasma membrane (Logeat et al., 1998). Upon ligand binding, endocytosis from the signal-sending cell (Wang and Struhl, 2004) provides a pulling force necessary to expose the S2 cleavage site for an extracellular protease, ADAM metalloprotease/TNF-α converting enzyme (Meloty-Kapella et al., 2012, Musse et al., 2012). After S2 cleavage, the signal-sending cell endocytoses the ligand with the remaining NECD, and the S3 cleavage in the signal-receiving cell by the intramembrane γ-secretase complex releases the NICD from the transmembrane domain (TM) (Levitan and Greenwald, 1995, De Strooper et al., 1999, Struhl and Greenwald, 1999). The NICD released from cell membrane translocates to the nucleus where it interacts with the CSL (*CBF1* in humans, *Suppressor of Hairless* in *Drosophila*, *Lag-1* in *Caenorhabditis elegans*) transcription factor complex, resulting in subsequent transcriptional regulation of target genes (Artavanis-Tsakonas et al., 1995).

Apart from the signals passing between neighbouring cells by *trans*-activation, the ligand-receptor interactions of Delta-Notch can occur within the same cell, resulting in functionally neutralized Notch receptors. This process is known as *cis*-inhibition (De Celis and Bray, 1997, Micchelli et al., 1997). Consequently, cells with high Delta levels turn into signal-sending cells and cannot receive signals via Notch (Sprinzak et al., 2010), giving rise to a unidirectional signalling mode during lateral inhibition termed the “walkie-talkie” model (Sprinzak et al., 2010, Sprinzak et al., 2011). Cis-inhibition is also important for the mechanism of dorsal-ventral boundary formation in the *Drosophila* wing disc (Del Alamo et al., 2011).

The third major component unpacked out of the segmentation clock is the system of gradients extending along the anterior–posterior axis of the PSM and thought to provide positional information in the tissue. The first pathway identified was FGF signalling (Dubrulle et al., 2001, Sawada et al., 2001), which was joined shortly thereafter by Wnt (Aulehla et al., 2003, Aulehla and Herrmann, 2004) and Retinoic acid signalling (Diez Del Corral et al., 2003, Moreno and Kintner, 2004). Those findings provided an initial solution to where and when PSM cells are allocated to segments.

Modelling has played an important role in understanding vertebrate segmentation, partly because oscillators have a long history of study from a number of theoretical perspectives. A first, simple model to describe the cyclic wave pattern across the PSM appeared as a supplement to the paper that described *c-hairy1* (Palmeirim et al., 1997). Subsequently, the three major genetic elements of the clock mentioned above have been gathered together in increasingly complex models that can recapitulate the cyclic wave pattern (Cinquin, 2007, Tiedemann et al., 2007, Morelli et al., 2009, Hester et al., 2011), and thus demonstrate the plausibility and sufficiency of this basic molecular framework for the segmentation clock. However, the field is a long way from understanding the system; many of the assumptions in these models have not been tested, and new components with vital roles are still being added.

One other property also less explored is the robustness of the segmentation clock. Unlike mammals and birds, poikilothermic animals, such as most teleost fish and arthropods, face temperature fluctuation from the environment during development. For example, zebrafish embryonic growth has a high temperature dependency (Kimmel et al., 1995). In addition, teleost somitogenesis frequency can have a 3-fold difference across a 10-degree temperature range (Fernandes et al., 2006, Schröter et al., 2008). However, the total segment number or somite length reveal only minor or no changes over these temperature ranges (Brooks and Johnston, 1994, Ahn and Gibson, 1999, Galloway et al., 2006, Schröter et al., 2008). These findings suggest a general property in teleosts that somitogenesis precisely compensates for the variation of elongation rate caused by temperature fluctuation, ensuring that the same body proportions are produced. How this occurs is not understood, and comparative studies across evolutionally distinctive species could be potentially insightful.

Vertebrates are not the only group to form repeated modules in their body plan, and metamerism has been identified in three major metazoan clades: annelids, arthropods and chordates. With the discoveries in the past decades from various arthropod species, it can be argued that the sequential addition of segments from a posterior zone is the primitive mechanism of segmentation from their common bilaterian ancestor. However, there are vast diversities in the genetic details of the segmentation process, even just within insects, suggesting complicated gain and/or loss of features of segmentation during arthropod evolution. On the one hand, we can appreciate the diversity of ways that animals can segment their bodies, and on the other, it would be difficult to directly determine homology from the molecular and genetic mechanisms. Nevertheless, from a systems-level, we may be able to classify some basic principles of segmentation shared between vertebrates and arthropods, whether it arose from convergent evolution or homologous traits (Richmond and Oates, 2012).

In this review, we aim to discuss vertebrate segmentation with particular emphasis on the role of the Notch intercellular signalling pathway. First, we present a brief introduction to the clock and wavefront model and then expand it into three tiers of biological length-scales: single cell oscillators, local synchronization (where Delta-Notch signalling plays key roles), and global control of the timing and pattern in the tissue. For the vertebrate part, we tend to present evidence from zebrafish (*Danio rerio*) because of our personal expertise, but also because much of what we know about the role of Notch signalling in the process has been learned in this animal. In each tier, we review both historical milestone discoveries and the latest concepts in the vertebrate field, and try to use these perspectives to review recent findings in arthropods. In Section 4, we discuss a relatively new concept of the wave pattern and Doppler effect on the timing of segment formation, and recent evidence on the effect of Delta-Notch signalling in the wave pattern. In Sections 5, 6, we then focus on more conceptual ideas about Delta-Notch signalling and its role in synchronization and lateral inhibition. In the concluding Section 7, we summarize and list several interesting open questions in the field.

## 2 The clock and wavefront model

**Fig. 2:**
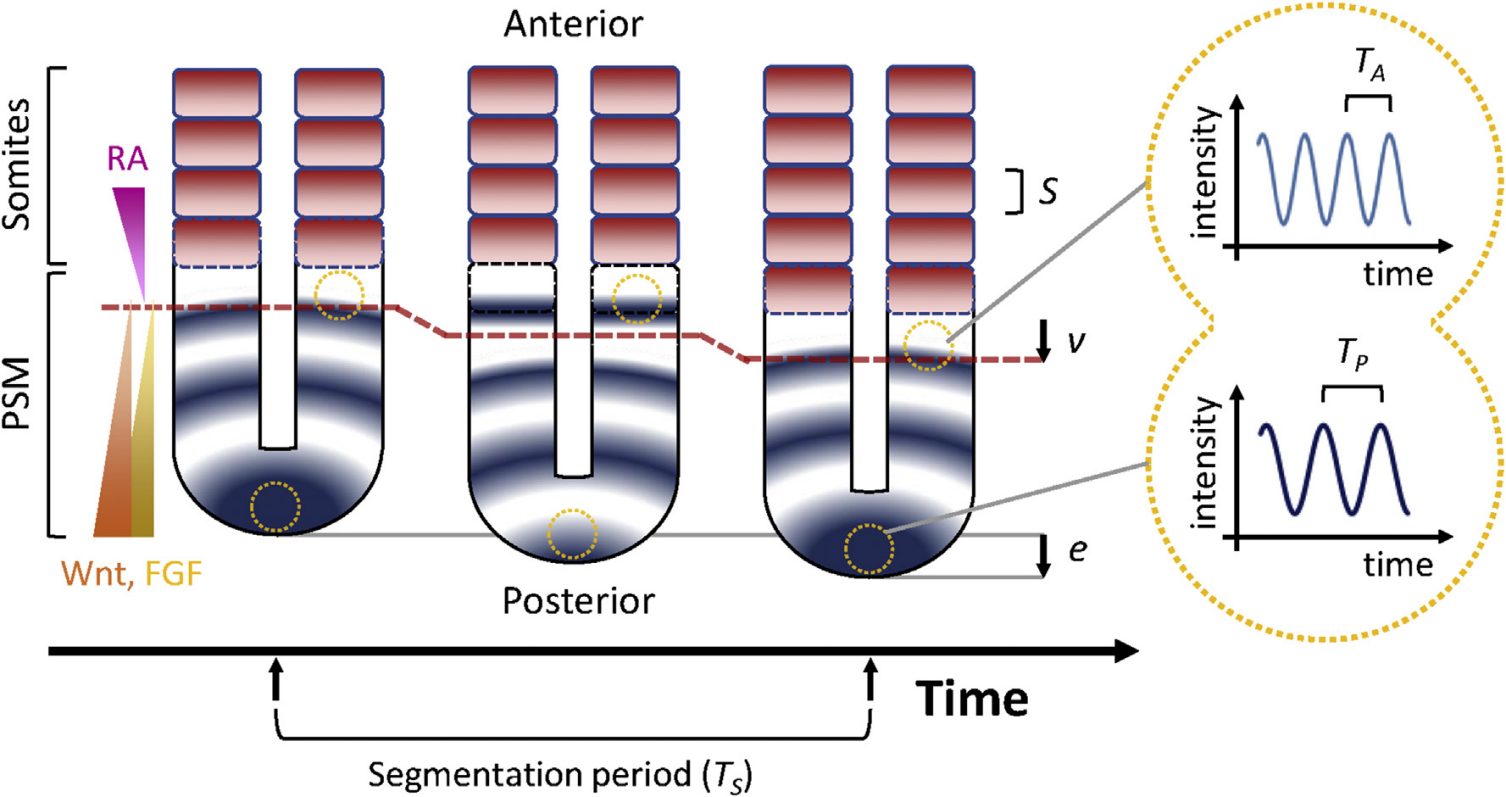
A schematic diagram of the vertebrate segmentation clock. In vertebrates, the wavefront (red dashed line) is influenced by Fgf and Wnt signalling gradients from the posterior end and a counter-gradient of RA from the somites. As the new somite forms and axis elongates, the wavefront sweeps posteriorly in concert. When the cyclic expression waves of *hes*/*her* genes (blue colour in the PSM) moves across the wavefront, the oscillation arrests and a new segment boundary is determined. Several measurable parameters are essential for analysing the clock and wavefront model, i.e. *S*, the segment length; *v*, the wavefront velocity; *e*, axis elongation rate. The segmentation period *T*_*S*_ is measured by the duration of each segment formation. By measuring the expression of *hes*/*her* in real-time at the anterior or posterior end of PSM over time (yellow dotted circles in the PSM), the oscillation can be revealed (yellow dotted inset at the right panel) and the anterior period *T*_*A*_ and the posterior period *T*_*P*_ can be measured. Note that *T*_*A*_ can be different to *T*_*P*_, see Section 4 and Fig. 5 for more details. Blue or black dotted boxes, nascent somites.

How is the temporal information from biological oscillators converted into organized spatial patterning of somites? Theorists have developed several simplified models that aim to reduce the complexity of vertebrate segmentation to its core mechanism. One of the earliest, and most dominant conceptual models is the clock and wavefront model proposed by Cooke and Zeeman (1976). In the original model the clock was a population of synchronous cellular oscillators, and the wavefront was a smoothly moving front of sudden cellular change that supplies longitudinal positional information. The wavefront interacted with the clock as it moved across the population. Cells changed their behaviour based on the phase of their oscillation at the time they passed the wavefront. This interaction produces a spatially periodic succession of cellular states, with one segment defined by the number of cells passing the wavefront in one cycle of the oscillator. Concomitant or subsequent changes in cellular adhesion, shape and arrangement would finally convert the periodic pattern to morphological segments. In the original model, the oscillators were assumed to be synchronous across the entire PSM, whereas following the discovery of *c-hairy1* we now know that although the oscillators show local synchrony, they are organized so as to form waves across the tissue (Fig. 2). From the initial observation, it was also proposed that the wave patterns repeat precisely with each forming segment (Palmeirim et al., 1997), and for a perfectly repeating pattern the entire tissue will oscillate with a well-defined period (Morelli et al., 2009); thus despite the waves, the PSM tissue still beats with a single rhythm.

According to this model, the relationship between length and time scales that follows can be expressed in a simple mathematical formulation. The segment length *S* is proportional to the speed of the wavefront *v* and the period *T* of the oscillation: *S* = *v* × *T*. Thus, an increase in segment length can result from an increase in the period of the clock, or an increase in the speed of the wavefront, for example. Moreover, an increased number of somites along the body axis can result either from a shortened period and/or an extended total segmentation time. The difference between the wavefront velocity *v* and PSM elongation rate *e* defines the PSM shrinkage/extension rate (Fig. 2).

Note that the clock and wavefront model expresses the simple hypothesis that *T*, above, is the period of a hypothetical genetic clock that determines the rate of morphological segment formation *T*_*S*_ (Fig. 2 bottom). Since the introduction of live reporters of the clock, it has become possible to investigate the waves in more detail (Masamizu et al., 2006, Aulehla et al., 2008, Niwa et al., 2011, Soroldoni et al., 2014, Shih et al., 2015). The arrival of each cyclic expression wave indeed marks the formation of a new segment, such that *T*_*S*_ is identical to the period of the clock in the anterior PSM, *T*_*A*_ (Fig. 2, right panel). However, in the zebrafish *T*_*S*_ has been measured to differ from the posterior period, *T*_*P*_ (Fig. 2, right panel), indicating that the segmentation clock does not beat with a single rhythm and the traditional clock metaphor may be too simple (Soroldoni et al., 2014). These differences in period arise as a consequence of the waves, and we will return to this topic in Section 4, below. Nevertheless, the clock and wavefront model has provided a powerful conceptual framework to interpret experimental results (Gomez et al., 2008, Gomez and Pourquié, 2009, Schröter and Oates, 2010) and is independent of any specific genetic network. Thus it has become the most accepted conceptual model for vertebrate somitogenesis so far.

## 3 A three-tier model of the segmentation clock

**Fig. 3:**
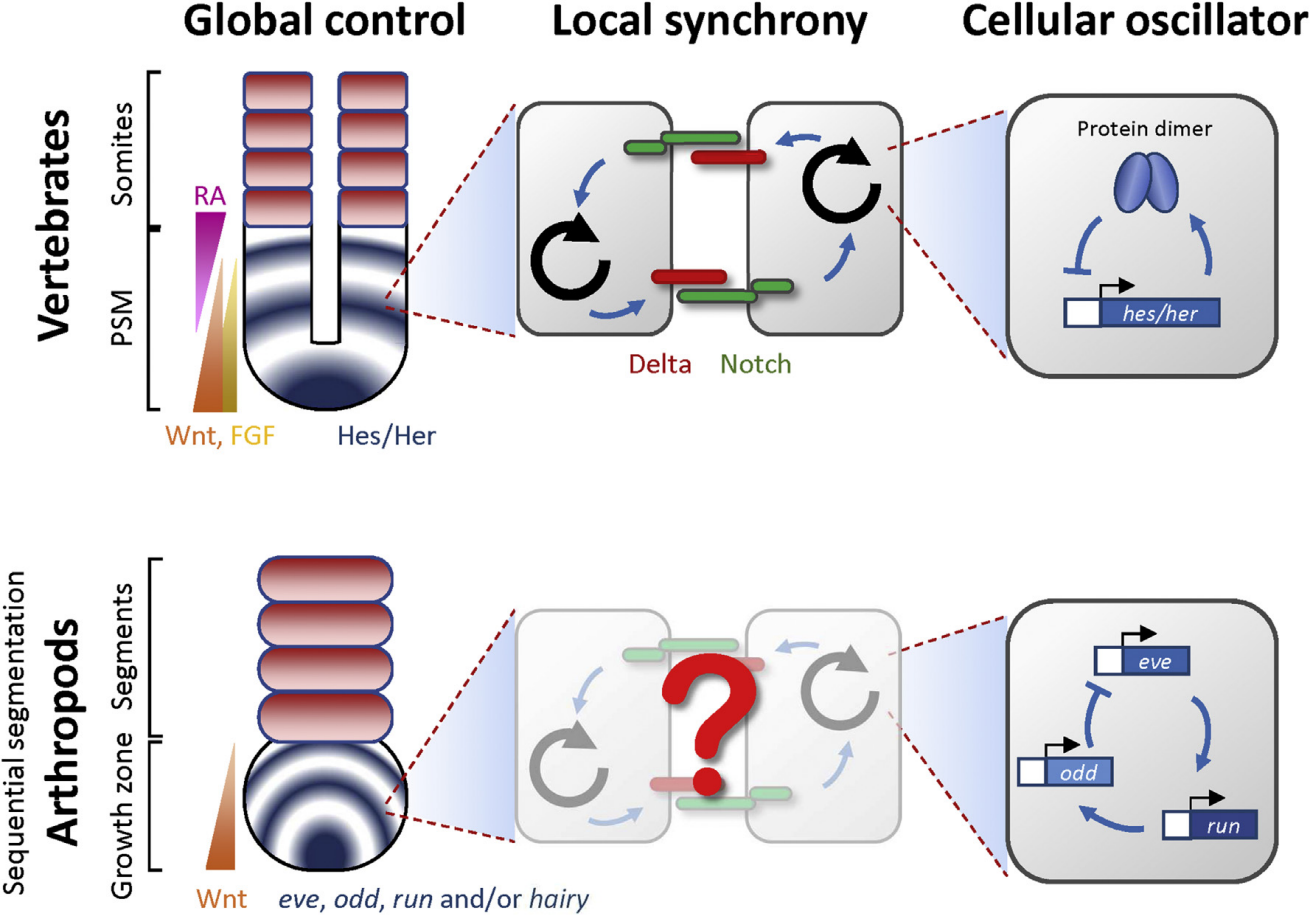
Comparing the three-tier model in vertebrates and sequentially segmenting arthropods. The segmentation clock can be dissected into three different scales. In the bottom tier (right panel), the cellular oscillator consists of a negative feedback genetic loop. The second tier (middle panel) is the local synchrony provided by intercellular signalling that sends and receives modulating signals to and from the core oscillators. Note that the local synchrony components can either express in cyclic/oscillating fashion, e.g. zebrafish *deltaC*, or in more static manner, e.g. zebrafish *deltaD*. The top tier is the global control (left panel) that defines where the oscillation should stop, where cells become determined, and produces the frequency profile across the unsegmented tissue. For the sequentially segmenting arthropods, we used the most well-studied model organism, *Tribolium*, for the comparison.

We have previously proposed a three-tier model to dismantle the segmentation clock into 3 different scales: single cell oscillators, local synchronization, and global control of the timing and pattern (Oates et al., 2012) (Fig. 3). This model provides a basic framework for biological multicellular oscillators (reviewed by Webb and Oates, 2016). To compare vertebrate, annelid and arthropod segmentation, Balavoine (2014) also proposed a model that contains three modules; in this case, anterior/posterior axis elongation, segmental periodicity, and segment polarity. For the sake of clarity, we point out that the processes described in the three-tier model of the vertebrate segmentation clock here largely correspond to the events described in the segmental periodicity module in Balavoine's model.

### 3.1 Cellular oscillator through a negative feedback loop

The basic units of most biological clocks are cells, in which the major clock components are usually genes (or more precisely gene expression and function) (reviewed by Rensing et al., 2001). An autorepression feedback genetic circuit provides a simple way to generate oscillations (Fig. 3, right panel).

Vertebrate *hes*/*her* gene homologues belong to the basic-helix-loop-helix (bHLH) superfamily, which are known as DNA-binding transcription factors appearing as homodimers (Leimeister et al., 2000, Trofka et al., 2012) or heterodimers (Sasai et al., 1992, Ma et al., 1994, Alifragis et al., 1997, Iso et al., 2001, Schröter et al., 2012). From the analysis of the mouse *Hes1* promoter, Takebayashi et al. (1994) demonstrated that the Hes1 protein is able to bind its promoter and to repress its own gene expression. Similar autorepressory feedback loops leading to oscillations by *hes*/*her* genes in PSM cells were proposed in mice (Hirata et al., 2002, Hirata et al., 2004, Bessho et al., 2003, Hirata et al., 2004) and zebrafish (Holley et al., 2002, Oates and Ho, 2002). Thus, continuous on-and-off switching of gene expression gives rise to oscillations, and the time delays from gene transcription to protein translation to protein degradation of *hes*/*her* genes sets up the intrinsic period of the segmentation clock (Jensen, 2003, Lewis, 2003, Monk, 2003).

How can one identify one or more genes as components in a core oscillator circuit in a cellular clock? There are two general criteria: (i) removal of their functions will crash the clock (Bessho et al., 2001b, Oates and Ho, 2002); and (ii), modification of their properties will tune the frequency of the clock (Schröter and Oates, 2010, Harima et al., 2013). A core oscillator also has the property of autonomy, namely the information that determines the timing should be self-contained, and not be dependent on external signals. Although the involvement of Hes/Her genes suggests that this single-cell feedback loop might be sufficient for oscillations, it is possible that this circuit may oscillate only when receiving either rhythmic or noisy external stimuli. In this interesting case, the minimal oscillating system might be a circuit that spans two, or more, coupled cells. The gold standard to distinguish this would be to take a cellular oscillator into an isolated environment and monitor cyclic gene expression. Two pioneering studies from chick and mouse used dissociated PSM cells to observe cellular oscillation without cell–cell communication (Maroto et al., 2005, Masamizu et al., 2006). However, lacking a live reporter in chick or a large set of quantified oscillation data, the extent to which these cells could sustain their oscillations *in vitro* remains uncertain. Recently, Webb et al. (2016) provided the first direct evidence that single cells from the zebrafish segmentation clock carrying a *her1-yfp* transgene can oscillate autonomously. Strikingly, the individual cellular oscillators were less precise and had a longer period than their counterparts in the embryo, suggesting additional roles for intercellular signalling *in vivo*. Whether cells from the segmentation clock of other species are self-sustaining in isolation, or whether they require signals from neighbours remains an open question.

**Fig. 4:**
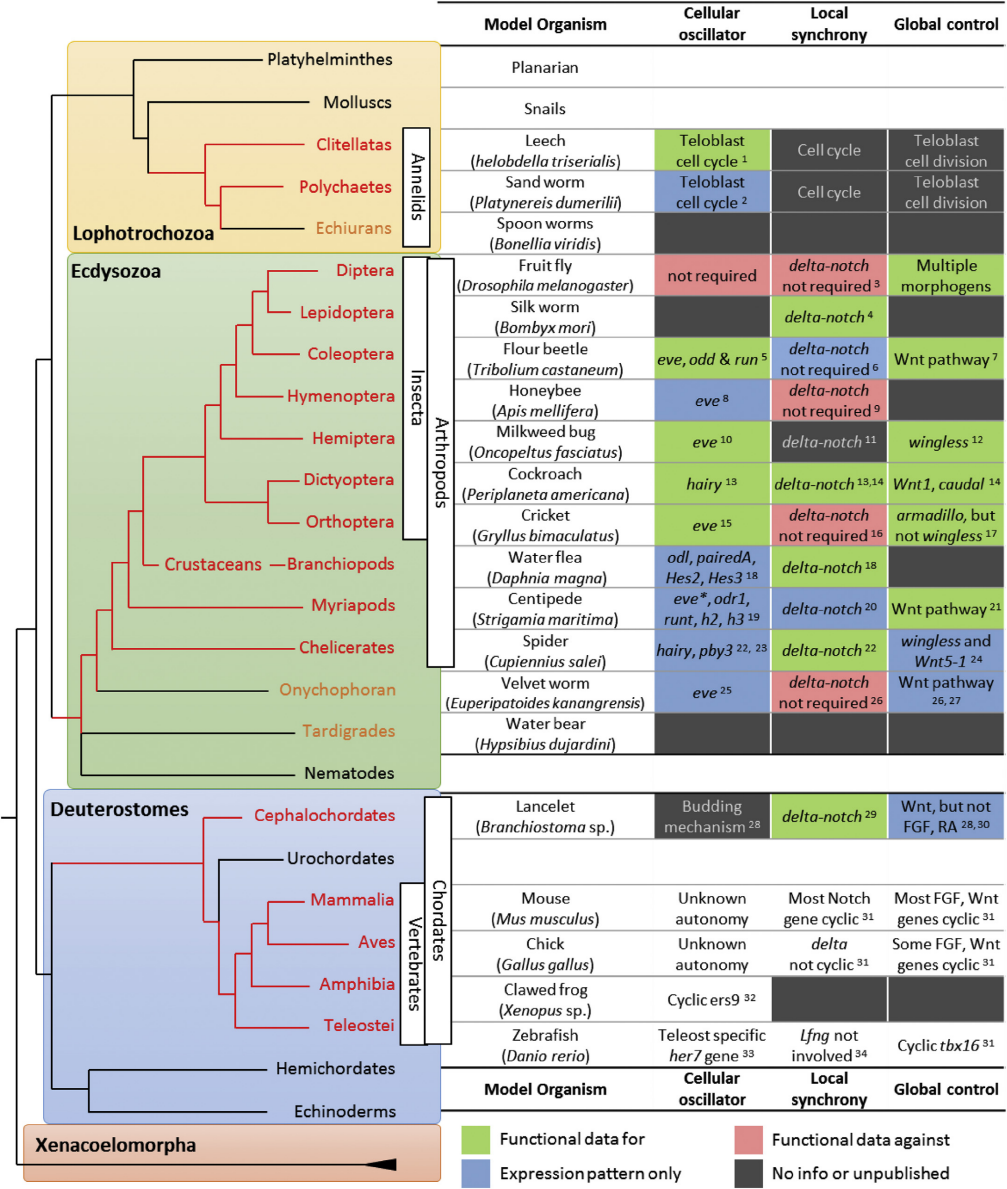
Similarity and diversity in the three-tier model in arthropod and vertebrate segmentation. The left panel depicts the phylogenetic tree of bilateria (Martin and Kimelman, 2009, Philippe et al., 2011, Liu, 2013, Cannon et al., 2016, Rouse et al., 2016) with the four major clades, Lophotrochozoa, Ecdysozoa, Deuterostomes and Xenacoelomorpha highlighted with different coloured boxes. In each clade, except for the Xenacoelomorpha, we list several representative phylogenetic classifications, in which names labelled in red are known for segmented bodies. Echiurans, onychophorans and tardigrades (orange) have partial or less pronounced segmentation. Red branch lines are connection of the segmented classes to their common ancestor branch points. The branch lengths are arbitrary. The table in the right panel lists the representative organisms in each phylogenetic group and their corresponding properties in the framework of the three-tier model (see Section 3). In the annelids, arthropods and cephalochordates, the lists are the known gene components or pathways that have the potential function corresponding to the three-tier model colour-coded in each cell as follows: green (functional data for), blue (expression pattern only), red (functional data against), or dark grey (no information or unpublished data). The criterion for the “functional data for” is whether a loss-of-function assay causes segment defects. Unlike most other arthropods groups, *Drosophila* segments nearly simultaneously, which is believed not to use a segmentation clock. For the vertebrates, we list selected discoveries that are different to vertebrate sister groups for each representative organism. *eve*, *even-skipped*; *odd*, *odd-skipped*; *run*, *runt*; *odl*, *odd-skipped-like*; eve*, *even-skipped1* and *even-skipped2*; *odr1*, *odd-skipped-related1*; *h2*, *hairy2*; *h3*, *hairy3*; *pby3*, *pairberry-3*; Superscripted numbers are references as 1. Weisblat and Kuo (2014); 2. Gazave et al. (2013); 3. Peel et al. (2005); 4. Liu (2013); 5. Choe et al. (2006) and Choe and Brown (2009); 6. Aranda et al. (2008); 7. Bolognesi et al., 2008, El-Sherif et al., 2014 and Oberhofer et al. (2014); 8. Binner and Sander (1997); 9. Wilson et al. (2010); 10. Liu and Kaufman (2005); 11. Chipman (2015); 12. Angelini and Kaufman (2005); 13. Pueyo et al. (2008); 14. Chesebro et al. (2013); 15. Mito et al. (2007); 16. Kainz et al. (2011); 17. Miyawaki et al. (2004); 18. Eriksson et al. (2013); 19. Brena and Akam (2013) and Green and Akam (2013); 20. Chipman and Akam (2008); 21. Hayden et al. (2015); 22. Stollewerk et al. (2003); 23. Damen et al. (2005); 24. Damen (2002); 25. Janssen and Budd (2013); 26. Janssen and Budd (2016); 27. Hogvall et al. (2014); 28. Schubert et al. (2001); 29. Onai et al. (2015); 30. Bertrand et al. (2015); 31. Krol et al. (2011); 32. Li et al. (2003); 33. Gajewski et al. (2006); 34. Prince et al. (2001).

In arthropods, several candidate genes of a core oscillator have been proposed. The pair-rule gene orthologs, e.g. *eve* (*even-skipped*), *odd* (*odd-skipped*), *run* (*runt*) and *hairy*, have been reported with a wave-like striped expression pattern in the growth zone in most arthropods studied (Fig. 4). In addition, some other gene families were reported, such as *delta* in cockroach and centipede (Brena and Akam, 2013, Chesebro et al., 2013) or *caudal* in centipede (Chipman and Akam, 2008). However, in the absence of real-time transgenic reporters of expression, conclusions were reached on the basis of carefully-staged mRNA expression patterns and knowledge of cell movement in the tissue. Functional studies by RNAi knockdown of *Tribolium hairy* showed no obvious phenotype in trunk segmentation even though *hairy* expresses in a cyclic-like pattern in the growth zone (Choe et al., 2006, Aranda et al., 2008). The flour beetle *Tribolium castaneum* is the most well-studied arthropod model species with sequentially adding segments. Choe et al. (2006) and Choe and Brown (2007) suggested a repressor negative feedback loop between *eve*, *odd* and *run* (Fig. 3, bottom right panel) as the primary oscillators, and the *odd* was found to oscillate under two-segment periodicity (Sarrazin et al., 2012). Two-segment periodicity is common is insects because the homology of the hierarchical network of pair-rule genes (Green and Akam, 2013). Indeed, *Drosophila*, which is thought not to have a segmentation clock, yielded the first example of two-segment periodicity: one domain of pair-rule gene expression demarcates two morphological segments. Intriguingly, the centipede shows both double and single segment periodicity depending on the axial position. From the analysis of mRNA expression patterns, two out-of-phase stripes of *eve1* and *delta* generate intercalated waves during two-segment periodicity stage (Brena and Akam, 2013, Valentin and Oates, 2013). In contrast, from the analysis of *pairberry-3* expression, the spider *Cupiennius salei* was suggested to be running under single segment periodicity (Schoppmeier and Damen, 2005).

Combined, this evidence allows one to speculate that arthropods might have undergone a transition of adapting an auto-repressor loop (one-segment periodicity) into a hierarchical pair-rule repressor loop (two-segment periodicity) during the evolutionary transition of ancestral insects, whereas vertebrates conserved an auto-repressor loop from the *hairy and Enhancer of split* gene family as the core oscillator (Peel et al., 2005). However, more direct evidence of which genes are the core oscillators in various arthropod species is required to test this hypothesis.

### 3.2 Local coupling by intercellular signalling

Individual cellular oscillators generate unavoidable noise from stochastic gene expression and degradation of mRNA and Protein (Jenkins et al., 2015, Webb et al., 2016). Thus, in order to achieve tissue level synchrony, cell–cell communication is essential to couple cellular clocks and to prevent their phases from drifting away. In the mid 1990's when the vertebrate homologues of *delta*-*notch* genes were discovered, the Notch signalling pathway was identified as being involved in somitogenesis (Bettenhausen et al., 1995, Conlon et al., 1995). Zebrafish mutants affecting somite formation were reported from the Tübingen screen (Van Eeden et al., 1996), among them several Notch pathway genes were also found (Holley et al., 2000, Holley et al., 2002, Itoh et al., 2003, Julich et al., 2005b). Since the cyclic stripe patterns of *hes*/*her* genes in the PSM are disrupted from loss-of-function Notch signalling, a straightforward explanation would be that Notch signalling is a part of the core component of the oscillators, or that Notch signalling triggers the oscillation (Holley et al., 2000). A major new role for Notch signalling in somitogenesis was proposed by Julian Lewis' laboratory (Jiang et al., 2000) – an interpretation called the desynchronization hypothesis: “*the essential function of Notch signalling in somite segmentation is to keep the oscillations of neighbouring presomitic mesoderm cells synchronized.*” Based on this point of view, a genetic network for vertebrate somitogenesis was proposed: *hes*/*her* family genes oscillate autonomously via transcriptional auto-repression; *hes/her* repressors also target one or more genes in the Delta-Notch pathway bringing their expression into the feedback-loop; rhythmic Delta-Notch signalling between PSM cells targets *hes/her* promoters causing them to be expressed slightly earlier or later than without the signal. Thus, genetic circuits in neighbouring cells are synchronized (Jiang et al., 2000, Lewis, 2003, Horikawa et al., 2006, Riedel-Kruse et al., 2007).

In zebrafish, *deltaC* is cyclically expressed along posterior–anterior axis of PSM (Jiang et al., 2000, Mara and Holley, 2007), while static expression patterns are found for *notch1a* and *notch2* in PSM (Oates et al., 2005a). Unlike *deltaC*, *deltaD* is found dynamically, but not cyclically expressed in the anterior PSM (Holley et al., 2002). Four *notch* and five *delta* paralogs have been identified in zebrafish so far. In mammals, two *notch* and two *delta* are required for somitogenesis as well, although the cyclic *delta* genes in zebrafish and mammals are not orthologs (Huppert et al., 2005, Pourquié, 2011). This implies that one cyclic *delta* plus one non-cyclic *delta* might be essential for securing the robustness of synchronization.

What are the phenotypes from Delta-Notch signalling loss-of-function and how are they interpreted? First, eliminating or reducing Notch signalling by either genetic mutation or pharmaceutical treatment results in disrupted somite boundaries and disordered *hes*/*her* gene expression in mouse (Ferjentsik et al., 2009, Kim et al., 2011), in chick (Dale et al., 2003) and in zebrafish (Jiang et al., 2000, Lewis, 2003, Horikawa et al., 2006, Riedel-Kruse et al., 2007), but not complete absence of somites nor loss of all *hes*/*her* gene expression.

Secondly, the segment defects seen in Delta-Notch mutants are always in the posterior part of the axis, or in other words, are late on-set phenotypes. In zebrafish, *notch1a*, *deltaC*, *deltaD* and *mind bomb* homozygous mutants still produce, on average, normal segment boundaries for the first 5–10 somites and then undergo an apparently sudden transition to producing defective segment boundaries that persists for the remainder of the axis (Van Eeden et al., 1996, Oates et al., 2005b, Zhang et al., 2007). These phenotypes can be quantified by the anterior limit at which defects are observed. This anterior limit of defects can be altered by applying dosage series or time course of the small-molecule γ-secretase inhibitor, DAPT, which blocks Notch ICD cleavage. Over time, the spatial wave patterns of the cyclic genes become more and more disordered, and this can be modelled as a decay process of the cellular oscillators gradually losing synchrony between neighbouring cells (Riedel-Kruse et al., 2007). The synchrony dynamics of PSM cells could be inferred by recording the axial positions of defective somite boundaries (Riedel-Kruse et al., 2007). Moreover, this apparent desynchronization is reversible: The PSM cells gradually lost synchrony while the coupling was blocked, and after washing out the DAPT and re-engaging cell–cell coupling, the cyclic gene wave patterns sharpened as the oscillators gradually restored synchrony. In arthropods and annelids, a type of segmental anomaly, termed helicomery or “spiral segmentation”, was frequently observed in trunk regions (Leśniewska et al., 2009), and multiple developmental defects were suggested to be associated with. The genetic causes of helicomery haven't been identified; thus, testing whether Notch signalling is involved in the formation or helicomery is still an open question.

Importantly, Delaune et al. (2012) showed by tracking *her1-yfp* transgene expression in zebrafish Delta-Notch mutants, that cells in the PSM continue to oscillate, but with a decrease in synchrony. Combined with the single cell autonomy data mentioned above, these results indicate that at least in zebrafish, Notch signalling doesn't drive, but instead modulates the timing of *hes*/*her* gene oscillation leading to the synchronization of neighbouring PSM cells.

Finally, the segmentation period can be altered by perturbation of Notch signalling. In zebrafish, a 20% longer segmentation period was observed in the anterior somites in *deltaD* and *mind bomb* mutants and in DAPT-treated embryos (Herrgen et al., 2010). Conversely, the segmentation period could be shortened about 6.5% in transgenic line, *Damascus*, carrying multiple copies of *deltaD-yfp* (Liao et al., 2016). In zebrafish, more Notch signalling speeds the clock up, and less slows it down. In *Nrarp*^−/−^ knockout mice, which have higher notch activity due to lack of *Nrarp* to down-regulate NICD activity by direct interaction, less somites were produced within the same duration (Kim et al., 2011). The authors estimated the clock period was about 5% longer. In the same study, a shorter segmentation period was detected by blocking Notch signalling with γ-secretase inhibitor. In mouse, more Notch signalling slows the clock down, and less speeds it up. From theoretical work, it was known that the collective period of coupled autonomous oscillators can be varied from the average period of individual oscillators, if there is non-neglectable time delay in the coupling (Schuster and Wagner, 1990). Thus, the opposite responses of zebrafish and mouse can be explained by the relative time between the signalling time delay and the intrinsic period of cellular clock, and this effect is amplified by the input of notch signalling strength (Lewis, 2003, Morelli et al., 2009, Oates et al., 2012). This alteration of timing via altered Notch-mediated coupling in segmentation clock may be a source of heterochrony during evolution. Although Notch signalling may have additional influences on period (Section 4), these data support the idea that Notch signalling is acting in vertebrate segmentation primarily via the synchronization of oscillators.

In the sequential segmenting arthropods, studies on silk worm (Liu, 2013), cockroach (Pueyo et al., 2008, Chesebro et al., 2013), brine shrimp (Williams et al., 2012), water flea (Eriksson et al., 2013), centipede (Chipman and Akam, 2008) and spider (Stollewerk et al., 2003) suggest Delta-Notch signalling is involved in body segmentation. However, studies on honeybee (Wilson et al., 2010) and cricket (Kainz et al., 2011) provided functional evidence that Notch is not required in these species, even though the *delta* genes are expressed in wave-like/stripe patterns. There seems no obvious evolutionary trend while organizing the information above on the arthropod phylogenetic tree (Fig. 4), and it is difficult (for us) to judge whether this is because of real diversity or simply insufficient evidence. One reason for this uncertainty might be because of the conserved interaction network between Notch and Wnt pathways on tissue growth in arthropods (Chesebro et al., 2013). RNAi knockdown of either these pathways usually causes the loss of posterior body structures, which make investigating Delta-Notch function in segmentation more challenging. How to disassociate segmentation and body elongation in order to look at the right time and/or place will become a high priority open question for future studies.

Although, in this review, we discuss the roles of Delta-Notch in arthropods in the section on local synchrony, studies from arthropods usually interpret Delta-Notch as the primary oscillatory genes driving downstream *hairy* or other pair-rule genes expression as an output (Pueyo et al., 2008, Chesebro et al., 2013; and reviewed by Graham et al., 2014). In *Tribolium*, neither the *delta* expression pattern (Aranda et al., 2008), nor functional studies suggest the requirement for Notch in segmentation (unpublished, cited in Aranda et al., 2008). However, tracking labelled cell clusters in the growth zone over time shows that the cells rearrange actively during segmentation, and most clusters of labelled cells will be distributed across at least two segments (Nakamoto et al., 2015). This finding suggests that the cell mixing distance is comparable to or longer than the wavelength of the pattern and, in the absence of an active synchronization mechanism, this would be expected to be a strong source of noise that would disrupt the coherent pattern, even if the oscillators always started in sync in the posterior end. Moreover, the expression pattern of the *hairy* gene is disturbed, but not lost in embryos deficient for Notch or Delta in spider (Stollewerk et al., 2003), implying that Delta-Notch signalling might serve a similar function as in vertebrates. Nevertheless, future studies are still required to test this hypothesis. To date, the synchronization mechanism for maintaining local spatial coherence in gene expression hasn't been proposed for arthropods (see Section 5 for more discussion).

### 3.3 Global control by signalling gradient to determine frequency profile and wavefront

The two main features of the oscillations in vertebrate PSM tissue are the existence of waves of gene expression, and the arrest of the waves at a defined point in the anterior. How are these features generated and regulated? Are they linked, or are they independently controlled? Work in the field has previously focused on the question of the arrest of the waves, in other words: where and how do the oscillations stop? This is thought to be closely related to the location of the wavefront, the position in the PSM where the cellular oscillators become committed to a stable segmental fate. It's a generally accepted hypothesis that the wavefront is determined by the combined action of signalling gradients: a combination of Wnt and fibroblast growth factor (Fgf) synthesized in the tailbud and highest in the posterior PSM, and a counter-gradient of retinoic acid (RA), synthesized in the formed somites and highest in the anterior PSM (reviewed by Aulehla and Pourquié, 2010) (Fig. 3, left panel). Thus, the wavefront corresponds to a threshold level in these signalling gradients where the cells exit their oscillating progenitor state, and begin to express the components of segmental fate. Because of the dynamics of axis elongation extending the tissue posteriorly, and segment formation shortening the tissue from the anterior, the moving sources of signals produce the wavefront velocity with respect to the formed somites (Fig. 2).

In thinking about this model, it is important to consider the steady input of cells into the posterior of the PSM from the tailbud, and the repeated exit of cells in newly formed somites from the anterior of the PSM. Over developmental time the PSM is not a fixed group of cells, rather the cells are continuously replaced in a flow from posterior to anterior. The signalling gradients introduced above therefore act in and on a moving cellular field. This has consequences for the effects of diffusion of ligands from sources at either end since the cells move away from one and towards the other. Furthermore, ligands that are bound to cells or ECM will be moved with the cellular flow. Lastly, internal cellular states, such as levels of mRNA that are loaded by synthesis in the posterior, will be transported across the tissue with the flow (Dubrulle et al., 2001, Aulehla et al., 2003, Gaunt et al., 2003). If these mRNAs are then translated into protein, such as is the case for FGF (Dubrulle and Pourquié, 2004), the source of the ligand itself becomes extended across the tissue. Thus, the distribution of signalling in the PSM will be given by some as yet unknown combination of the diffusive and advective processes at work in the tissue.

How the oscillating cells use the signalling gradients to arrest their oscillations is still an active area of research. Perturbations to the gradients can be challenging to interpret solely terms of segmentation because Wnt, Fgf and RA also play critical roles in vertebrate embryonic elongation and posterior body development (Wilson et al., 2009). Transient or restricted perturbation has therefore been key to dissecting roles in segmentation. As an example, we focus next on the evidence for a role of Wnt in the regulation of a wavefront in vertebrates, as posterior Wnt signalling is also thought to play an important role in arthropods.

Wnt3a mutant mice lack caudal somites, have a disrupted notochord, and fail to form a tailbud (Takada et al., 1994); likewise, zebrafish with early knockdown of wnt3 and wnt8 display a strong perturbation to elongation and lack most posterior structures, including somites (Shimizu et al., 2005). Gradients of Wnt signalling activity marked by nuclear beta-catenin have been reported across the mouse and zebrafish PSM, with highest levels in the posterior (Aulehla et al., 2008, Bajard et al., 2014). Ectopically active Wnt signalling throughout the mouse PSM changes the position of oscillator arrest in the anterior, creating a longer PSM (Aulehla et al., 2008, Dunty et al., 2008), and implantation of Wnt3-overexpressing cell clusters in the PSM of chick shifts the locally-forming somite boundary anteriorly (Aulehla et al., 2003). Transient up- or down-regulation of Wnt signalling in the zebrafish PSM gives rise to a run of shorter or longer segments, respectively, in the absence of changes to body elongation or to segmentation period (Bajard et al., 2014). These changes to segment length were prefigured by a corresponding shift in the spatial expression domains of differentiation markers and cyclic genes. From these studies has emerged a model whereby posterior PSM cells are maintained in an undetermined, oscillating state by elevated levels of Wnt signalling and the wavefront is triggered as cells in the PSM move below a concentration threshold in the Wnt activity gradient (Aulehla et al., 2003, Aulehla and Herrmann, 2004, Bajard et al., 2014, Mallo, 2016). How the Wnt threshold is interpreted by the oscillating cells as a stop signal, and how the Wnt gradient interacts with Fgf and the opposing RA signal gradient remain open questions.

In contrast to the control of where oscillations stop, much less is known about how the wave patterns are regulated. In order for oscillators to form a wave pattern, they must have a spatial profile in their phases. A stadium wave offers a simple analogy – spectators must stand or sit at a time slightly offset to their neighbours along the stadium to make a travelling wave. The phase profile of the cellular oscillators in the vertebrate PSM is proposed to arise from a frequency profile of oscillators gradually slowing their cycles as they take on more anterior positions (Kaern et al., 2000, Giudicelli et al., 2007, Morelli et al., 2009). Recent work has observed this slowing in the zebrafish PSM *in vivo* (Shih et al., 2015) and in mouse cell culture systems (Tsiairis and Aulehla, 2016). How the slowing is controlled and whether it can be regulated independently of the position of arrest is not understood, however. Plausible proposals involving both the tissue-level signalling gradients and local Delta-Notch coupling have been put forward (Murray et al., 2013), but not yet tested.

One promising line of investigation may be the role of transcription factors downstream of Wnt signalling, such as members of the *Cdx* family. Mutations in *Cdx* genes, like mutations impairing Wnt and Fgf signalling, cause posterior truncations and disturb axial patterning of the embryonic structures in mouse and zebrafish (Davidson et al., 2003, Chawengsaksophak et al., 2004, Young et al., 2009, Savory et al., 2011). In the flour beetle *T. castaneum*, Tc-Cad is expressed in a Wnt-dependent posterior gradient, whose anterior limit marks the anterior-most stripe of the cyclic expression of *Tc-eve* (El-Sherif et al., 2012). Perturbation of genes that regulate the level of Wingless activity showed that the Tc-Cad gradient was changed in position, slope and/or maximum value, and moreover, the *Tc-eve* stripe pattern was correspondingly altered (El-Sherif et al., 2014). These striking results suggest that spatial gradients of Wnt or Cad (or both) might be the biochemical mechanism of the frequency profile underlying the *Tc-eve* gene expression waves. Comparing the potential consequences of a gradual slowing of oscillations versus a sudden arrest using a simulation, the authors proposed that a frequency gradient might serve as a buffer against noise that would otherwise perturb precise boundary formation. Whether this is indeed the case, and what role *Cdx* genes may play in vertebrate segmentation needs now to be explored.

Regardless of the genetic mechanism(s) underlying their regulation, the existence of gene expression waves has a number of interesting consequences for the timing of segmentation, as will be discussed in the next section.

## 4 Wave patterns and their influence from Delta-Notch signalling

Gene expression waves are an obvious and beautiful phenomenon in the segmentation clock, but what is their consequence? Previously, conceptual and formal models have described segmentation under the simplifying assumption of steady state (Lewis, 2003, Riedel-Kruse et al., 2007, Morelli et al., 2009). Typically, this means that key parameters keeping track of, for example, the size of the tissue, the basal production rate of a key protein, the coupling strength, the shape of the frequency profile, etc., have been kept constant in order to simplify the analysis of dynamic variables that describe the activity of the oscillators, for example, phase, period, amplitude, concentration of cyclic gene product, etc. For a given set of parameters, this gives rise to a perfectly repeating segmentation clock generating an “infinite snake”. This assumption has proven powerful and useful, and may be well justified when considering relatively short developmental intervals. Theoretical results have shown that at steady state, the gene expression waves do not play an important role: the segmentation clock generates segments with the same timing period and length regardless of the number and shape of waves (Morelli et al., 2009, Ares et al., 2012).

However, over longer developmental times, it is evident that the segmentation clock does not repeat itself perfectly; the most obvious feature being that the oscillating tissue changes its length continuously and in all vertebrates it eventually shortens and disappears entirely when the last segment has been formed. More generally, there is no reason to think that each of the other parameters would be constant over longer developmental times, either. If these changes are very small or slow compared to the dynamics, they can be neglected in a model without loss, but when the changes are of a similar time scale to the dynamics, they must be considered explicitly. For example, when trying to model a transient experimental perturbation to the system, such as a heat-shock induced gene expression, changes to the affected parameters may drive changes in the system's dynamic output.

Even in the course of normal development, the shortening of the zebrafish PSM occurs at a rate that is high enough to affect the dynamics. In a rapidly shortening tissue, the presence of gene expression waves results in a Doppler effect at the anterior end as the tissue boundary moves into the oncoming waves (Soroldoni et al., 2014, Jörg, 2015). In the same way that the pitch of a sound is higher for a moving observer approaching a source than a static observer at the source, the period with which gene expression waves arrive at the anterior end of the PSM has been experimentally shown to be shorter than the period with which they leave the posterior (Soroldoni et al., 2014). This anterior period is the same as morphological segmentation, and so the Doppler effect contributes to determining the period of segmentation.

The Doppler effect is one consequence of gene expression waves in a segmentation clock that changes its length; another follows from longer-term changes in the phase profile across the tissue, a phenomenon termed the Dynamic Wavelength effect. For more details, we refer the reader to Jörg (2015). In summary, the output segmentation period observed at the anterior border of the PSM will depend on the period of oscillations set by the genetic feedback circuits visible in the posterior PSM, yet can be significantly modified by tissue-level contributions from wave effects. Importantly, the magnitude of the Doppler contribution is determined by the velocity of tissue shortening and the wavelength of the wave pattern encountered at the anterior end of the PSM (Jörg, 2015).

**Fig. 5:**
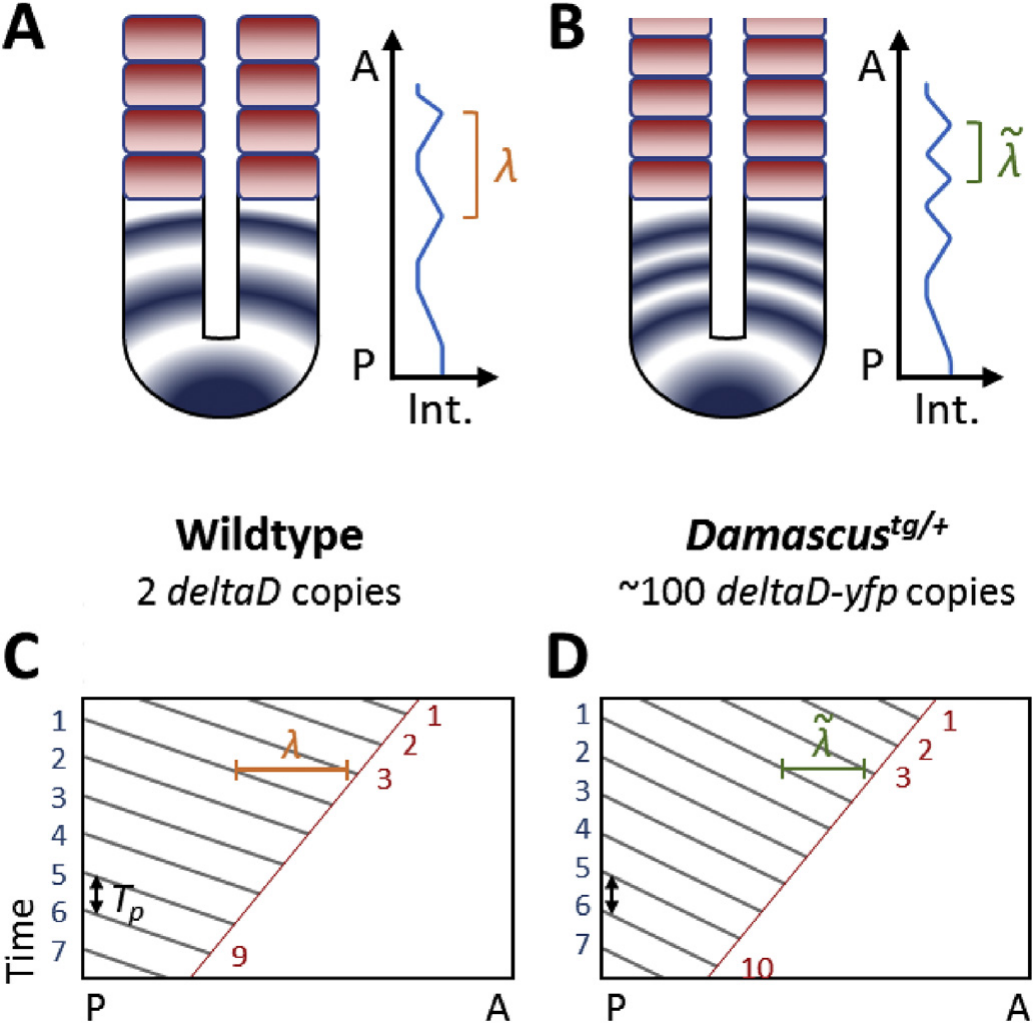
Delta-Notch signalling speeds up segmentation by altering the wave pattern. (**A and B**) A schematic diagram of the altered wave pattern and wavelength in a zebrafish transgenic line, *Damascus*, which carries approximately 100 *deltaD-yfp* copies. *Damascus* showed faster somitogenesis and more segments within the same segmentation duration. Cyclic expression of *her* gene waves are shown as blue colour in the PSM. The most anterior wavelengths (λ and $\tilde{\lambda }$) can be measured by *her* gene inter-stripe length from the plots of averaged intensity profile (right panels). (**C and D**) Kymographs of the PSM over developmental time to explain the faster somitogenesis found in *Damascus*. The grey lines in the kymographs are peaks of *her* gene expression waves, and the red lines connect where and when new somite boundaries form. The blue numbers indicate how many waves starting from the posterior end, with the posterior period, *T*_*P*_, can be measured by peak intervals. The red numbers label how many new segments are generated within the given time window. The anterior period, *T*_*A*_, can be calculated from the duration divided by the number of segments formed. The anterior end (red) numbers are larger than the posterior end (blue) numbers, i.e. *T*_*A*_ < *T*_*P*_ because the shorter wavelength in the anterior PSM in *Damascus* increases the contribution of the Doppler effect. This yields the smaller *T*_*A*_ found in (D) with identical *T*_*p*_ to (C). For simplification, potential contributions from the dynamic wavelength effect are not drawn in the kymographs. Please refer to Soroldoni et al. (2014) and Liao et al. (2016) for more details. A, anterior; P, posterior; Int., intensity.

The discussion above focuses on the effect of wave phenomena in setting the segmentation period during normal development, but these arguments also predict that the segmentation period can be changed from wildtype values by altering the magnitude of the wave effects. As mentioned above in Section 3.2, the zebrafish *Damascus* transgenic line carries approximately 100 *deltaD-venus*/*yfp* transgene copies and showed higher Notch signalling strength and a faster segmentation rate in the embryo (Liao et al., 2016). The PSM length decreases at the same rate as in wildtype, but the anterior wavelength of the *her1* gene expression waves in *Damascus* is about 20% shorter than in wildtype. This change in wavelength predicts a segmentation period due to a change in the contribution of the Doppler effect that is shorter than wildtype (Fig. 5), and in quantitative agreement with the segmentation period measured in *Damascus* by time-lapse microscopy.

As discussed above in Sections 3.2, 3.3, the wave pattern is typically classified under the regulation of global control, while Notch signalling is thought primarily responsible for local synchrony. Thus, our finding suggests Notch signalling not only synchronizes oscillators, but is also capable of globally altering the wave pattern, thereby changing the segmentation period. Alteration of wave patterns through changes in oscillator coupling has been observed in several theoretical studies (Murray et al., 2011, Ares et al., 2012), but how Notch-dependent patterning works in the PSM at a biochemical level is still unknown and may involve changes to the coupling strength and time delay (Morelli et al., 2009, Liao et al., 2016).

One corollary from the existence of wave effects is that the segmentation period could potentially be altered relatively quickly without changing the period of the underlying genetic oscillations. A recent study on *Tribolium* discovered that the estimated segmentation period is not constant and could have up to a five–fold difference from one two-hour interval to the next (Nakamoto et al., 2015). Interestingly, the authors concluded that the anterior–posterior length of each segment is about the same. If we interpret this information with the clock and wavefront model, it suggests that the wavefront velocity of *Tribolium* is not constant and must be coordinated with segmentation clock period in order to produce constant segment lengths. If this was the case, an internal modulation of the period of the cellular oscillators is one possibility, but a change in the Doppler contribution from the changing tissue length could be a key factor. Nevertheless, the quantifications in this study are based on the ordering of static snapshots and thus the contributions from individual variation and/or flexibility are masked and mixed (Love, 2010). Direct *in vivo* measurement of the key parameters of the oscillating tissue will be crucial to untangle this intriguing phenomenon.

## 5 Is local synchrony dispensable in arthropod segmentation?

As discussed above in Section 3.2, it is not clear whether the local synchrony function maintained by Notch signalling in vertebrates is required in arthropods. There are three hypotheses that may explain the apparent lack of requirement for Delta-Notch mediated synchronization in flour beetles and other arthropods. First, some intercellular signalling pathway other than Notch may synchronize oscillators in the posterior. Because the phase information has to be passed from one cell to its neighbours, juxtacrine signalling pathways would be good candidates. At present, no other substitute pathway has been proposed or identified.

Secondly, other mechanisms could rescue a partial loss of synchrony during boundary formation. Waves of cyclic gene expression may not have to arrive at the wavefront with all neighbouring cells perfectly synchronized to be able to form normal sharp boundaries – a critical threshold may suffice (Riedel-Kruse et al., 2007). This rescue of boundary formation from an imperfect patterning input could be achieved by one or more distinct mechanisms. For instance, mechanical tension may straighten the cell–cell border as is the case in the *Drosophila* wing disc dorsal-ventral boundary, or cell adhesion differences may produce repulsion as in vertebrate hindbrain rhombomere formation, or extracellular matrix may be deposited into the forming somite furrow and act as a scaffold (reviewed by Minelli, 2000, Dahmann et al., 2011). Tests of these hypotheses would first require analysis to determine the relationship between boundary integrity and oscillator synchrony *in vivo*, which remains a challenging open question in vertebrates.

Finally, maintenance of local synchrony may be not essential, and the coherence of gene expression waves may have instead been built in to the upper or lower tier. One extreme example from outside of arthropods is the segmentation of the central nervous system in the leech *Helobdella*, which is a cell lineage-driven process controlled by the cell cycle of teloblast stem cells that pile up segmental precursor cells sequentially from posterior (Weisblat, 1985, Weisblat and Shankland, 1985; reviewed by Weisblat and Kuo, 2014). In this case, the pattern coherence (synchrony) is maintained by the cell cycle, which is also the cellular oscillator. Interestingly, one of the arthropods, the malacostracan crustacean *Parhyale hawaiiensis* segments its epidermis in a similar manner (Hannibal et al., 2012), suggesting this teloblast-like mechanism isn't unique to recent annelids. The other example is the well-known case of *Drosophila* segmentation. Despite the quite distinctive mechanism of segmentation used by *Drosophila*, the spatial precision of one or more gap gene boundaries in the fruitfly is thought to be initially conferred by global controls, i.e. signalling gradients across the body, like *bicoid* or *caudal* (Gregor et al., 2007). However, whether the sequentially segmenting arthropods share homology with *Drosophila* or even annelids to bypass the need for vertebrate-like active synchronization is unknown. Isolated PSM cells are noisy oscillators with a much lower precision than observed in the embryo (Masamizu et al., 2006, Webb et al., 2016), suggesting that oscillators are coupled strongly against noise *in vivo*. We have discussed the case where the mixing of oscillators occurs at length scales greater than the local pattern wavelength and is deleterious for the maintenance of the pattern. However, cell mixing over length-scales similar to or shorter than the local pattern wavelength may facilitate global synchronization by intercellular signalling by dispersing clusters of oscillators trapped in locally synchronized patches (Uriu et al., 2012, Uriu et al., 2014). It will be insightful to investigate the interplay of noise level and cell movement in arthropod models.

## 6 The many faces of Notch signalling: lateral inhibition and synchronization

Phylogenetic analyses of eukaryotic genomes suggest that a functional Notch pathway emerged in ancestral metazoans, and some regulatory genes, e.g. *notchless*, appeared as early as in a eukaryote common ancestor (Gazave et al., 2009). Despite many biological activities being linked with Notch signalling (Fig. 1), its function can be still simplified as making cell fate decisions and then patterns.

**Fig. 6:**
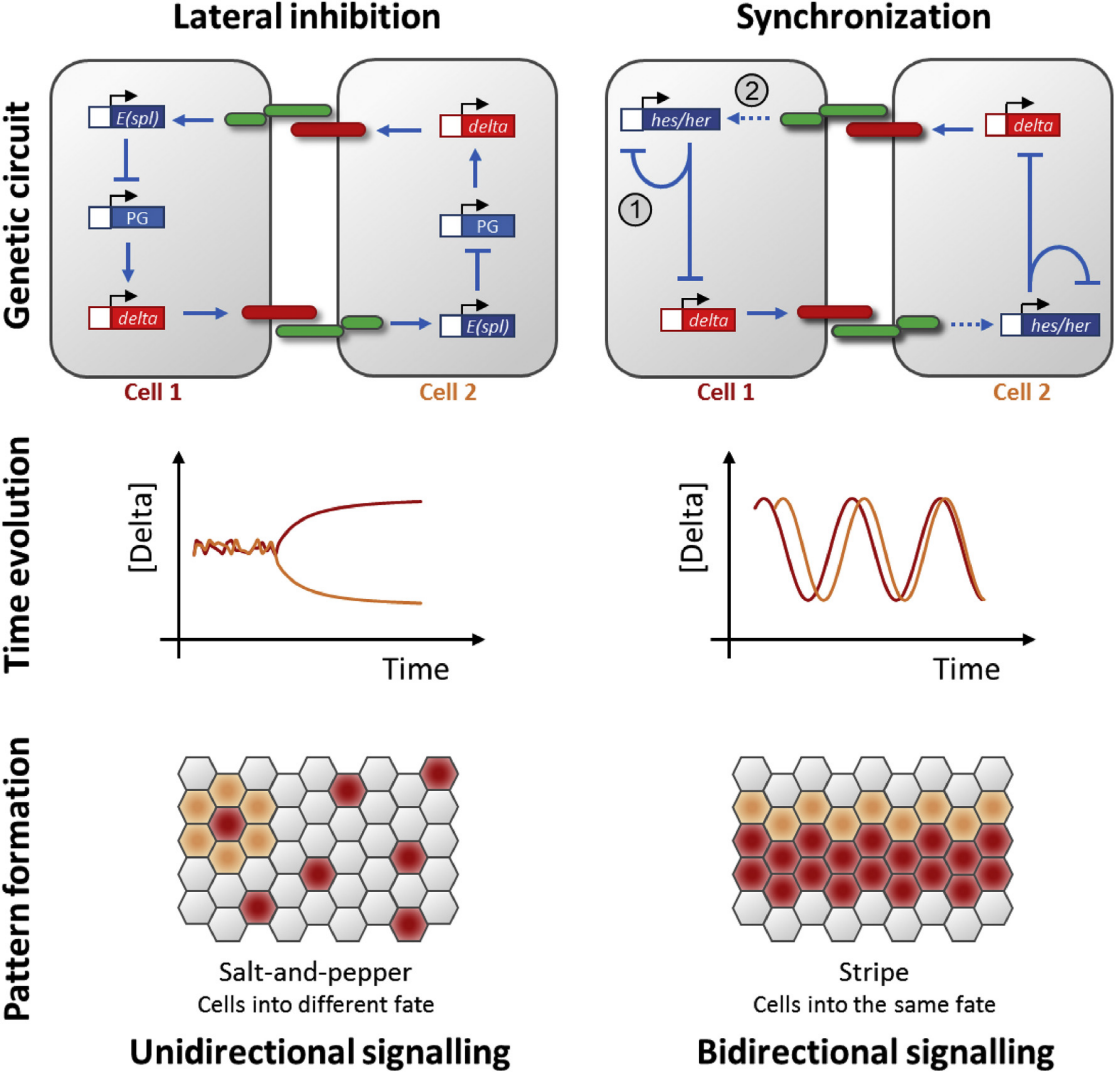
A speculative evolutionary transition from a lateral inhibition to synchronization circuit. A schematic representation of lateral inhibition and synchronization via Delta-Notch signalling. (**Top panel**) The minimal genetic circuits of lateral inhibition (left) and vertebrate somitogenesis synchronization (right) using zebrafish as an example. Circled numbers 1 and 2 point out the two proposed transitions from the lateral inhibition to the synchronization circuit. See Section 6. (**Middle panel**) time evolution of the Delta concentration from the 2-cell model above. In lateral inhibition, one cell inhibits the Delta expression from the other after a short fluctuation. In the synchronization scenario, the two cells oscillate in phase over time. (**Bottom panel**) the conventional patterns generated in the population of cells level show opposite outputs in lateral inhibition (local differences) and synchronization (local similarity). Cells are depicted as hexagons. Cells with high Delta expression are shown in red, and the cells in orange are examples of interacting cells as described above. *E(spl)*, *enhancer of split*; PG, proneural gene; dashed line with arrow, positive modulating input.

One intriguing question is how Notch signalling accomplishes diverse outputs, like lateral inhibition (Collier et al., 1996, Lewis, 1998), border formation (Kageyama et al., 2008, Oginuma et al., 2010) or synchronization (as discussed in Section 3.2). Among them, the outcome of lateral inhibition, which uses unidirectional signalling, and synchronization, which uses bi-directional signalling, are intuitively opposite to each other (Fig. 6). Lateral inhibition produces a fine-grained, “salt-and-pepper” pattern of differing identities in neighbouring cells, whereas synchronization produces a longer-range coherence in neighbouring cell states. This becomes yet more interesting when the same major biochemical components are found in both scenarios, for example, zebrafish *deltaC*, *deltaD*, *notch1a* and mouse *Hes1* (Haddon et al., 1998, Holley et al., 2000, Holley and Takeda, 2002, Kageyama et al., 2007). While the existence of multiple tissue-specific or genetic context-dependent components might be an obvious answer, on the other hand, it's also appealing to search for one simple explanation.

Canonical Notch signalling is a relatively short pathway, with no second messenger involved, but various modifications or co-factors are able to modulate the signalling outcome. This complex array of possibilities can be usefully investigated by mathematical modelling. In models, Notch signalling can be described as simply as one Delta ligand and one repressor to successfully recapitulate lateral inhibition patterning (Formosa-Jordan and Sprinzak, 2014). Most simulations are modified versions of the original lateral inhibition model (Collier et al., 1996) with one or more ligands, receptors and downstream components. Modulators and co-factors of Notch pathways have often been represented as time or strength parameters in the models. By tuning these parameters, several potential routes for switching between fine-grain and uniform patterns have been predicted. Two examples for such time-related parameters are provided here. In the delayed coupling theory of segmentation oscillators, when the signalling time delay is close to half of the intrinsic oscillator period, synchronized oscillators could be trapped into anti-phase pattern via cell–cell coupling (Morelli et al., 2009, Herrgen et al., 2010, Shimojo and Kageyama, 2016b). Alternatively, when a time delay is introduced to lateral inhibition models, synchronous oscillation could be observed before cell fate determination happens (Veflingstad et al., 2004, Glass et al., 2016), thereby securing the fidelity of lateral inhibition (Glass et al., 2016). For strength-related parameters, two examples are provided. A modified lateral inhibition model (*sensu* Collier et al., 1996) with noise suggests that the stable solution of homogeneous pattern appeared only with low strength of interaction between neighbouring cells (Reppas et al., 2016). During chick inner ear development, low signalling strength from *Jag1* ligand alone induces *Hey1* expression in a fine-grain pattern during prosensory development, while *Jag1* and *delta1* combined with a higher strength to generate a fine-grain pattern of *Hes5* when hair cells are determined (Petrovic et al., 2014, Petrovic et al., 2015).

The question of pleiotropic signalling is not only an interesting and appropriate topic for modelling approaches, but also has a fundamental evolutionary aspect, especially for the arthropods that have only one *notch* and one *delta* gene. Pueyo et al. (2008) and Couso (2009) suggests a three-step gradual model of evolutionary morphological transition, beginning from a salt-and-pepper lateral inhibition pattern to the alignment of selected cells in a row, to patterning of lines or boundaries, to “run-away” patterning waves. Despite the morphological similarities in the intermediate steps, the alignment in rows and the line/boundary pattern might require extra molecular inputs to be feasible. For example, the dorsal-ventral boundary of the *Drosophila* wing imaginal disc requires the spatial distribution of *Fringe* in the dorsal part to create a restricted sensitivity of the *Notch* receptor to *Delta* and *Serrate* ligands, which are also expressed in a spatially restricted manner (De Celis et al., 1997, Irvine, 1999, Fortini, 2009). Without spatial definition of the signals, the output may revert back to a salt-and-pepper pattern as shown in cell culture experiments (Lebon et al., 2014). Furthermore, to generate waves by moving these previously established boundaries might need the coordination of global spatial controls. Thus, modification to the topology of genetic circuit would be essential to accomplish these steps. In contrast, a confluent 2D culture of PSM cells generate wave-like patterns of synchronized oscillating *Lfng* reporter expression, suggesting that the moving waves in the PSM may be self-organized and not require external spatial signals (Tsiairis and Aulehla, 2016).

Despite the differences in tissue architecture and timing of the various patterns, from the perspective of signal transduction, lateral inhibition and synchronization circuits share high similarities (Lewis et al., 2009, Soza-Ried et al., 2014) (Fig. 6). A speculative evolutionary model for transforming from a CNS-type lateral inhibition to a zebrafish PSM-type synchronization circuit is proposed here with two key steps: (1) establishing an autorepressor feedback loop with Notch target genes; and (2) removing or diminishing the dependency of the target gene from Notch signalling (Fig. 6).

The first step is to combine the genetic functions of the *E(spl)* and proneural genes, which act successively to turn a Notch signal into a loss of Delta expression, into feedback repression of the *E(spl)* locus and direct repression of Delta. This direct repression would significantly reduce the time delay from E(spl) expression to switching off the Delta signal. Molecularly, this could be achieved via several potential paths. For example, a mutation in either the ancestral *E(spl)* or proneural gene coding regions could create a novel heterodimer, which adopts both inhibitory functions. The bHLH gene family is known for promiscuous dimerization and thereby producing various regulatory functions (Kageyama et al., 2007, Schröter et al., 2012), suggesting that this is evolutionarily feasible. It could also happen by gene duplication and/or rearrangement of *E(spl)* related genes, which is suggested to occur with high frequency during evolution (Dearden, 2015), and subsequent modification of repressor binding sites in the regulatory region of one of the copies. The outcome of this step is a *E(spl)* gene that can oscillate under its own feedback control, while maintaining repression of Delta.

In the second step, the strength of the input signal from Notch has to be reduced from driving to modulating the expression of the proto-oscillator *E(spl)* gene in order to allow oscillations to persist without Notch. This step is not necessary to achieve a synchronization circuit per se, but is required to mimic the zebrafish example, where oscillations occur in isolated cells as discussed in Section 3.2. In the context of lateral inhibition, *hes*/*her* gene expression is known to be Notch-dependent, that is, the majority of expression is lost if Notch signalling is compromised. Examples include zebrafish *her4* in the developing CNS (Yeo et al., 2007), chick *Hes5* in the developing inner ear (Doetzlhofer et al., 2009, Petrovic et al., 2014), and the *Drosophila E(spl)* genes in the ventral embryonic epidermis (De Celis et al., 1996). Along with reduced Notch, the *E(spl)* proto-oscillator gene needs to acquire a direct positive input, potentially from a ubiquitous or tissue-specific transcriptional activator. The outcome of this second step is the transfer of activation of the *E(spl)* gene from Notch signalling alone to being regulated by other factor(s), allowing for autonomous oscillators that can be coupled.

What would an intermediate step look like? The anticipated circuit types would be a lateral inhibition run either by low Notch signalling strength or including oscillation. The former may be less likely given existing modelling results (Petrovic et al., 2014, Petrovic et al., 2015, Reppas et al., 2016). For the latter type, a clue could be found from the behaviour of mouse *Hes1*, which is known to oscillate in CNS neuronal progenitors under the influence of Notch signalling for several cycles before lateral inhibition fixes the neuronal fate (Shimojo et al., 2008, Kageyama et al., 2015). Interesting, within a cell, *Dll1* (*Delta-like1*) and *Hes1* oscillate in anti-phase with their targets the proneural genes, *Ascl1* and *Neurog2*. At the tissue level, cells also oscillate out of phase, which is proposed to result from the length of the signalling time delay caused by *Dll1* expression (Shimojo and Kageyama, 2016a, Shimojo and Kageyama, 2016b). However, shortening the delay associated with Dll1 expression by experimentally removing *Dll1* introns did not produce the anticipated in-phase synchronized oscillation (similar to the PSM), rather it caused “oscillation death” (Shimojo and Kageyama, 2016a). These striking results may indicate that the window of time delay that allows sustained oscillation, either in- or out-of phase, is very narrow, or they may indicate that mechanisms other than the delay are at work.

Starting from the synchronized oscillators of the zebrafish PSM, Herrgen et al. (2010) obtained an uncorrelated, fine-grain pattern of oscillators by over-expressing the ubiquitin ligase *mind bomb* in zebrafish PSM. Mind bomb is required for the rate-limiting step of Delta internalization during Notch signalling (Itoh et al., 2003), and increasing Mind bomb levels was anticipated to shorten the signalling time delay. A caveat to the interpretations of this experiment is that the fine-grain patterns were measured from static gene expression patterns and not imaged dynamically. Thus, although much remains to be understood, these attempts to experimentally move from one pattern to the other by a one-step manipulation suggest that lateral inhibition may not differ greatly from synchronization in terms of genetic circuit components and topology. Consistent with this, we are not aware of any tissue that uses both types of mechanism during the same developmental interval; presumably cross-talk between the circuits would be too large for both to function. Interesting open questions in this topic include: (i) how do neuronal precursors escape from their oscillating state (Bonev et al., 2012), and (ii) how is the decision to use either a lateral inhibition or a synchronization circuit made and then carried out during development?

## 7 Open questions and outlook

Before the auto-repressor clock of *hes*/*her* genes was proposed as a model for segmentation, there were suggestions that vertebrate segmentation might be controlled by a pair-rule system similar to *Drosophila* due to the striped patterns of *hes*/*her* gene expression in the PSM (Muller et al., 1996, Yamaguchi, 1997). Interpreting broad gene expression stripes as evidence of a pair-rule mechanism was intuitive because of the well-established and elegant *Drosophila* example. Since more efforts were devoted to understanding the role of oscillations in vertebrate segmentation, the term “pair-rule” is no longer used in the field. Similarly, one should be cautious about interpreting broad gene expression stripes as cyclic expression without more direct evidence.

Overt segmentation defects are just one of the informative phenotypes when the segmentation clock is perturbed, and a careful and quantitative approach may be necessary to distinguish other subtler effects. For example, the zebrafish *hes6* gene does not have cyclic pattern of mRNA expression and the *hes6* mutant is homozygous viable and fertile with apparently normal segments at standard lab culture temperature. Without further analysis, it might have been concluded that *hes6* has no function during segmentation. However, the Hes6 protein shows cyclic levels, the *hes6* mutant has a 6% longer segmentation period that can be measured by time-lapse microscopy (and is reflected in 16 longer segments in the trunk, versus 17 in a wildtype sibling), and produces defective somite boundaries with low penetrance at low temperature (Kawamura et al., 2005, Schröter and Oates, 2010). In explaining the change in period, a role for a “dimer cloud” of Hes/Her homo- and hetero-dimers in the zebrafish clock has been proposed (Schröter et al., 2012). Only a subset of these can bind DNA, but the formation of non-DNA binding dimers can change the stability and availability of the participating proteins, and thereby alter the dynamics of the circuit.

Takashima (Takashima et al., 2011) and Harima (Harima et al., 2013) generated a series of transgenic mice in which the three introns of the cyclic *Hes7* gene had been deleted individually and in combination. All these lines showed strong embryonic segmentation defects, and correspondingly perturbed axial skeletons that were similar to the phenotype of the null mutant (Bessho et al., 2001b). From this, the authors concluded that the splicing of introns contributes to the overall delay in the feedback loop. However, in addition, Harima noticed that in one of these lines, the somites of the neck still formed; indeed, they formed more quickly than in wildtype (Harima et al., 2013). Direct imaging of the *Hes7*-luciferase reporter *in vivo* confirmed that the segmentation clock ticked faster during formation of the neck somites, before subsequently becoming disorganized. This careful analysis revealed the first example of a segmentation clock ticking faster than normal, but the issue of how to generate oscillations that are both faster and stable remained.

A final example is the zebrafish *deltaD* gene, which, as mentioned above in Section 3.2, does not show cyclic expression (Holley et al., 2002). The *after eight* phenotype was first described as a homozygous mutant with posterior segmentation defects beginning around the eighth segment (Van Eeden et al., 1996, Holley et al., 2000). This phenotype was one of the examples that led to the proposal of the synchronization function for Delta-Notch signalling (Jiang et al., 2000), and time-lapse recording of individual Her1-YFP reporter-expressing cells oscillating out of phase with their neighbours in the PSM of an *after eight* mutant brought direct evidence to support this long-standing hypothesis (Delaune et al., 2012). Multiple-embryo, time-lapse microscopy further revealed that the anterior, apparently normal segments were in fact longer and formed more slowly than in wildtype siblings, introducing the concept that coupling could modulate the period of a population of synchronized cells (Herrgen et al., 2010). Recently, a haplo-insufficiency effect of *deltaD* on coupling strength was discovered using a re-synchronization assay that quantifies how long the segmentation clock takes to recover from being fully desynchronized (Liao et al., 2016), and a transgenic line that over-expresses the DeltaD protein from its endogenous regulatory regions was shown to make segments at a consistently faster rate along the entire axis of the embryo (Liao et al., 2016). As discussed above in Section 4, this period phenotype is underlain by an alteration in the wave pattern of cyclic genes consistent with a change in the magnitude of the Doppler effect. Direct real-time imaging of the segmentation clock in this or a similar line will be necessary to better understand how Delta-Notch signalling orchestrates these changes.

To sum up, broken boundaries don't reveal very much about the dynamics inside the clock. The tool kit for analysing segmentation has become expanded and multifaceted and quantitative approaches are crucial. As new techniques and approaches are developed, genes and mutants reveal previously hidden phenotypes and our understanding of the segmentation clock grows richer and more nuanced. There are still many unanswered questions, and surely more surprises around the corner.

### 7.1 Technical challenges

In this final section of the review, we will discuss some synchrony-related technical challenges that are fundamental, yet haven't been conquered and so leave many questions unanswered.

#### 7.1.1 Measurement of phase

Development of an oscillator phase readout for microscopy would provide insights to many unsolved fundamental questions. Fluorescently protein tagged *hes*/*her* genes have initiated the first big step to our knowledge (Masamizu et al., 2006, Delaune et al., 2012, Soroldoni et al., 2014), but one drawback of a single tagged transgene is that the phase and amplitude information are mingled together in the time series. Currently, using mathematical tools to extract phase from short and noisy time series is difficult. One option is to use the wavelet transform, similar to a windowed Fourier transform, which generates a time-dependent phase signal and frequency spectrum. This requires at least one full oscillation cycle to extract the phase identity, and many more cycles would be required for reliable calculation using some standard variation of the Fourier transform (Webb et al., 2016). Thus, typical time series remain hard to analyse, and deciphering what an oscillator does in the final cycle as it stops is problematic. Double-tagged oscillators with shifted phases could provide phase information from the intensity ratio between the two tags, in a similar way to cell-cycle probes that independently label S-phase and M-phase (Sakaue-Sawano et al., 2008). In mouse, tags on components of Notch and Wnt pathways, which have been shown to oscillate in anti-phase (Aulehla et al., 2003, Dequeant and Pourquié, 2008) could potentially provide this information. One caveat is that it remains unclear whether these pathways are reporting from the same core oscillator circuit, or mark separate oscillators that can be coupled. In zebrafish, similar sets of genes oscillating in anti-phase have not been identified (Krol et al., 2011), therefore candidates could be the *her1* mRNA/Her1 protein pair, the Her1 protein/DeltaC protein pair, or combinations of weakly phase-shifted genes from the existing list of cyclic transcription (Krol et al., 2011). One immediate advantage of a phase readout is that the synchrony level of all oscillators in a defined 3D space could be measured from a relatively short time-lapse movie, or even a snapshot. Currently, estimating the level of synchrony is complicated by reporter gene amplitude variation and the difficulty of reliable long-term cell tracking *in vivo* in densely-packed tissues (Bhavna et al., 2016).

#### 7.1.2 Measurement of coupling strength and delay

Currently, estimation of coupling strength and delay is made using theory to infer the values from tissue-level dynamics (Herrgen et al., 2010). While this has been remarkably insightful, there are a number of assumptions built into the theoretical description. Definitions of coupling strength and delay appropriate for the question at hand must be chosen and direct measurements of their distributions must then be made. Although it's important to investigate these properties *in vivo*, the complexity and variability of the embryonic context remain formidable. An *in vitro* device suitable for measuring the time taken for two previously isolated oscillating cells to synchronize, in conjunction with a marker of Delta and/or Notch proteins that allowed the coupling delay to be observed, would enable direct measurement of coupling strength (Jörg et al., 2014). Synthetic biology studies using Delta-Notch gene tagged cell lines have shown that Notch signalling is capable of recapitulating *in vivo* tissue patterns in multicellular formats *in vitro* (Sprinzak et al., 2010, Matsuda et al., 2015, Morsut et al., 2016). Furthermore, primary culture studies of PSM cells also have proved the feasibility of oscillations *in vitro* (Maroto et al., 2005, Masamizu et al., 2006, Tsiairis and Aulehla, 2016, Webb et al., 2016). Presumably a well-designed micropattern (Desai et al., 2009) or microfluidic chip (Frimat et al., 2011) could allow one to measure synchronization or lateral inhibition with defined cell–cell contact area and numbers, even mimicking the spatial arrangement of oscillators favoured for simplicity in theoretical descriptions.

#### 7.1.3 Measurement of segment boundary defects

The integrity of segment boundaries is often used as the readout of a functional segmentation clock, and the presence of defects is indeed well correlated with a decrease in spatial organization of the oscillating cells in the PSM, at least in zebrafish (Jiang et al., 2000, Riedel-Kruse et al., 2007, Bajard et al., 2014). Because of the challenges of directly imaging the segmentation clock, the relatively high-throughput nature and reliability of using segment boundary defects as a key phenotype argue for their continued use. Nevertheless, we propose that three caveats to the interpretation of published results must be considered:

(i) Existing markers for boundary integrity may not reveal all defects. Defects have been routinely scored in either the initial somite boundary or in the later-forming chevron-shaped myotome boundary in various studies. Contrast has been achieved either in the light microscope, or using a range of different molecular probes. In zebrafish, the *xirp2a* riboprobe gives a robust and high contrast signal at the myotome boundary, and has been used reliably and quantitatively to compare results within and between laboratories over nearly 10 years (Deniziak et al., 2007). Nevertheless, there has been no systematic comparison of these methods, and it is not clear what defects may have been, or indeed are still being missed.
(ii) Boundary defects may not be equivalent. In perturbation experiments, boundaries have previously been scored as either defective or normal, and this binary approach has been successful in allowing behaviours to be quantified and described. However, closer inspection suggests that the strength of the defects appears to increase rapidly and non-linearly as the segmentation clock desynchronizes, for example. This can be seen in Fig. 1E, in Riedel-Kruse et al. (2007). A systematic method to measure, classify or rank segment defects would allow an additional level of sensitivity in the description of defects that may better report on dynamics or reveal new processes.
(iii) A segment boundary may change its integrity over time. As mentioned in (i) above, boundaries have been scored at different times in their development. While it has been implicitly assumed that a defective segment remains defective, and vice versa, there are examples that contradict this simplification. For example, in the *before eight* and *natter* mutants that disrupt integrin-fibronectin activity, somite boundaries are initially well-formed but are not maintained (Julich et al., 2005a, Koshida et al., 2005). Conversely, the anterior-most somites of the *beamter* mutant that lacks DeltaC protein are defective when initially formed, yet the myotome boundaries that develop from them a day later appear normal (Van Eeden et al., 1998, Herrgen et al., 2010). Furthermore, even in normal development, the segment–segment interfaces are a 3D structure whose shape is evolving in developmental time in an anterior to posterior progression (Rost et al., 2014). Clearly, there are multiple mechanisms intermediate between segmentation clock output and the final morphological boundaries of the myotome and sclerotome that will form the scaffold for locomotion, and more than one time point may need to be sampled to interpret the underlying activity of the segmentation clock.

While these caveats were motivated in terms of understanding the role of Delta-Notch signalling, they are relevant to interpreting any of the mechanisms in the segmentation clock. In general, the development some imaging-based order parameter that would allow segment boundary defects to be consistently classified will help to investigate the links between patterning processes, such as the synchronization state of the clock, and the resulting anatomy.

### 7.2 Flexibility and robustness

Vertebrate segmentation is a robust developmental process. In zebrafish, a wildtype animal scarcely makes a defective segment, and it also has stable segmentation period and segment number. In humans, vertebral defects occur at a frequency of 0.5/1000 to 1/1000 (Shands and Eisberg, 1955, Wynne-Davies, 1975), although the majority of these do not cause clinical problems. Notch signalling output is known for a high sensitivity to its input strength, analogous to a rheostat, and thus the biochemistry of the Notch pathway is said to provide high flexibility. How this flexibility is linked to the robust outcomes of patterning and morphogenesis at tissue level is an intriguing question. Félix and Barkoulas (2012) provided insight into this question by comparing organogenesis in closely related species of nematodes. They discovered that a fixed genotype-environment condition defined as the input could generate a striking range of variance in intermediate developmental phenotypes, but the system-level output was somehow buffered to secure the overall robustness of the final tissue architecture. This buffering however, is limited, and sufficiently strong perturbation to genotype and/or environmental factors will eventually lead to increased variance in system output. The range of a buffer zone represents the requirement for robustness in the tissue under the multiple challenges of mutation, environmental fluctuations, and noise in molecular processes. Similarly, the mechanism of buffering is expected to differ with the specific developmental context.

**Fig. 7:**
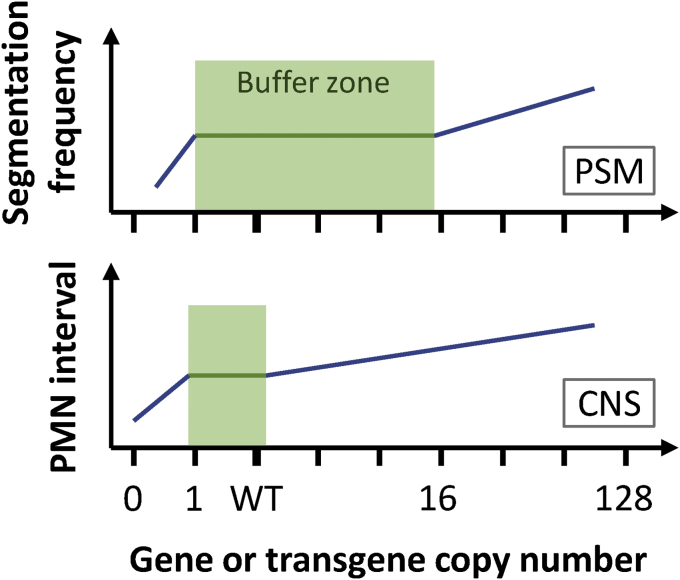
Different Notch signalling buffer zones in different tissues. A schematic representation showing the outputs responding to *deltaD* copy number changes in the PSM or CNS tissues. In the PSM, the output is the segmentation period, which follows from the magnitude of coupling. In the CNS, the output is the distance in the tissue between the primary motor neurons (PMN interval), which reflects the strength of lateral inhibition. Buffer zones (green high-lighted regions and horizontal green lines) are the copy number ranges in which the outputs showed no difference to wildtype (WT). Blue lines represent increased or decreased outputs from the WT level.

Our study of *deltaD* signalling levels in the zebrafish PSM and CNS also discovered similar phenomena, revealing different buffering capacity in the two tissues (Fig. 7) (Liao et al., 2016). At the gene expression level, DeltaD protein in the posterior PSM was well correlated to its gene copy number, but only copy numbers on the extremes of the range (the null mutant with zero copies and *damascus* with ∼100 copies) showed a change in segmentation period or segment boundary defects. All intermediate signalling levels showed wildtype development, defining a broad zone in which changes in signalling level were buffered into the same phenotype. In contrast lateral inhibition in the CNS is sensitive to all different copy numbers (except the difference between 1 and 2 copies), resulting in a very narrow buffer zone. Thus, these organ systems show a strong difference in phenotypic output “gain” across the range of DeltaD input signal. Since changes in DeltaD level affect the CNS more strongly than the segmentation of the body axis, these results predict an interesting evolutionary consequence of changes to Delta-Notch gene copy number, namely the evolutionary dissociation of these organ systems.

The buffering mechanisms at work are unknown so far. Is there a signalling modulator differentially expressed in PSM and CNS that actively tunes the sensitivity of Notch signalling to Delta input? To address this question, a preliminary step would require a reliable readout of Notch signalling in these tissues. One approach is to directly examine the amount of NICD (Huppert et al., 2005), and another candidate method would be to measure a sensitive and reliable Notch target gene, like *her4* (Yeo et al., 2007, Ozbudak and Lewis, 2008). Alternatively, the difference in the size of the buffer zones may follow from the system-level organizing properties of the lateral inhibition and synchronization processes; these properties may not depend explicitly on the levels of the molecules, and physical approaches may be needed to understand the buffering mechanism.

In addition to the requirement that segmentation frequency scales with elongation rate at different temperatures, the internal workings of the tiers of the segmentation clock must also be balanced across a wide temperature range. For example, how is the change with temperature in the time-scales of the cell autonomous Hes/Her feedback loop balanced with temperature changes in Delta-Notch signalling, which relies on orthogonal cellular processes such as vesicle trafficking in the Golgi and endocytosis? A study of the *Drosophila* mutant *rumi*, an O-glucosyltransferase that adds glucose to EGF repeats on the Notch extracellular domain, showed that multiple O-glucose residues on Notch maintained the ligand-dependent S2 cleavage at high temperatures (Leonardi et al., 2011). These findings provide a hint as to how Notch signalling buffers against changes in temperature, but how this is coordinated with the other tiers is not known. Likewise, how temperature sensitivity of the global signalling gradients is coordinated and integrated with the other tiers remains to be investigated.

### 7.3 Summary

In this review we have described the basic model for the organization of the vertebrate segmentation clock. In doing so we have tended to focus on zebrafish, but we emphasize that the vertebrates possess genetically distinct segmentation clocks, which may use variant mechanisms to achieve the overall embryonic function of sequential and rhythmic patterning. We discussed the evidence for current models that give Delta-Notch signalling a primary role within the segmentation clock as a means of synchronizing noisy, oscillating cells to a common local rhythm. Phase differences over tissue length-scales give rise to waves of gene expression in the oscillating tissue and we covered recent findings that, when the segmentation clock is not at steady-state, the waves play an active role in tuning the period of segmentation. Moreover, elevated coupling through Delta-Notch signalling can change this wave pattern, thereby further altering the segmentation period. It will be interesting to see whether any of these mechanisms are to be found amongst the arthropods. We proposed a scenario in which an ancestral Delta-Notch lateral inhibition circuit could evolve into a synchronization circuit of the type found in zebrafish, and discuss the differential response to changes in Delta-Notch signalling in organ systems. New technologies are rapidly changing our ability to investigate the role of Notch in segmentation, and we highlighted several open questions and a wish list of technical advances that might assist in this endeavour.
